# Sodium in the New Zealand diet: proposed voluntary food reformulation targets will not meet the WHO goal of a 30% reduction in total sodium intake

**DOI:** 10.1007/s00394-022-02864-5

**Published:** 2022-03-30

**Authors:** Nan Xin Wang, Sheila Skeaff, Claire Cameron, Elizabeth Fleming, Rachael Mira McLean

**Affiliations:** University of Otago, Dunedin Campus, Dunedin, New Zealand

**Keywords:** Food reformulation, Sodium, Salt, New Zealand

## Abstract

**Purpose:**

To simulate the potential impact of the HeartSAFE 2020 programme, a food reformulation initiative by the New Zealand (NZ) Heart Foundation, on sodium intake in the NZ adult population.

**Methods:**

A representative sample of NZ adults aged 15 years and older completed a 24-h diet recall survey, with 25% of participants completing a second diet recall, in the 2008/09 New Zealand Adult Nutrition Survey (*n* = 4721). These data were used to estimate sodium intakes of participants. The effect of altering the sodium content of 840 foods in 17 categories and 35 sub-categories included in the NZ HeartSAFE 2020 programme was simulated. The simulated sodium intake reductions in each food sub-category for the entire sample were calculated. Using sampling weights, simulated reductions in population sodium intake and by sociodemographic subgroups were also analysed.

**Results:**

Sodium intake from foods included in the HeartSAFE 2020 programme was 1307 mg/day (95% CI 1279, 1336) at baseline. After applying the HeartSAFE 2020 targets, potential sodium intake was 1048 mg/day (95% CI 1024, 1027). The absolute sodium reduction was 260 mg/day (95% CI 252, 268), corresponding to 20% sodium reduction for the foods included in the NZ HeartSAFE programme.

**Conclusion:**

Current sodium targets featured in the NZ HeartSAFE programme will not meet the 30% sodium intake reduction set out by the WHO Global Action Plan. A more comprehensive strategy consistent with the WHO SHAKE Technical Package is needed to advance the goal of sodium intake reduction.

**Supplementary Information:**

The online version contains supplementary material available at 10.1007/s00394-022-02864-5.

## Introduction

To combat the effects of non-communicable diseases, including cardiovascular disease (CVD), the World Health Organization (WHO) published its *Global Action Plan for the Prevention and Control of Noncommunicable Diseases 2013–2020*. One of the nine voluntary targets agreed by member states was to achieve “a 30% relative reduction in mean population intake of salt/sodium” by 2025 [1]. There is a strong dose–response relationship between sodium intake and elevated blood pressure [2], a leading risk factor for CVD [3]. As part of WHO efforts to assist governments to reduce population sodium consumption, of which salt is the primary source, *The SHAKE Technical Package for Salt Reduction* was published and includes a series of evidence-based policy options and interventions for salt reduction [4]. One of the suggested interventions is a recommendation to identify foods and food categories high in sodium, to set target levels for the amount of sodium in these foods categories, and then to progressively lower the sodium content of the foods within a specified time frame, a process known as food reformulation [4].

New Zealand (NZ) does not have recent data on population sodium intake. Over the last 25 years, only three studies have been published using the gold standard method of 24-h urinary sodium excretion and all carried out in convenience sample of adults. Those studies reported 24-h urinary sodium excretion was 3100 mg/day (*n* = 704) in 1998 [5], 3459 mg/day (*n* = 98) in 2011 [6], and 3386 mg/day (*n* = 299) in 2012 [7], consistently showing that the sodium intake of NZ adults exceeds the suggested dietary target of 2000 mg/day [8]. Furthermore, in the most recent study, McLean et al. reported that 77% (*n* = 229, 85% of men and 69% of women) of their participants exceeded the Upper Level of Intake (sodium recommendation in NZ before 2017) of 2300 mg/day of sodium [7]. Despite evidence of CVD being the leading cause of death in NZ [9], and its relationship with high blood pressure and a high sodium diet, the government does not have a comprehensive national sodium reduction strategy.

The NZ Heart Foundation, however, has established a national food reformulation programme known as HeartSAFE. This programme, in partnership with the food industry, has set voluntary targets to lower the sodium content of several high-volume, lower cost food products to achieve maximum public health gains [10]. In 2007, the Heart Foundation piloted salt reduction targets with breads (Project Target 450 [11]), and 1 year later, packaged loaf breads produced by main NZ companies reported an 18% sodium reduction in these breads [12]. In 2010, HeartSAFE was introduced to include more food categories and as of 2020, 17 food categories and 35 sub-categories have been assigned a voluntary maximum sodium target [13]. These food categories (including sausages, condiments, breakfast cereals, takeaway foods, and snack foods; see Table 2) have been chosen as they have been identified as being those that are of high sodium content and commonly consumed in NZ [14]. In particular, since the pilot programme, breads have expanded from packaged loaf breads to include artisanal breads, gluten-free varieties and flat breads. In this study, we simulated the potential impact of the HeartSAFE 2020 targets on sodium intake in the NZ adult population using the NZ Adult Nutrition Survey which was conducted prior to the introduction of HeartSAFE. Our aim was to test the scenario where all products complied with the HeartSAFE targets and simulate the impact this would have on population sodium intake. In particular, we wanted to test whether this scenario would result in the 30% relative reduction in population sodium intake recommended by WHO.

## Methods

This study involves secondary analysis of data from the 2008/09 NZ Adult Nutrition Survey (2008/09 NZANS). Data from all study participants (*n* = 4721) are used in this analysis.

### Study population

The 2008/09 NZANS [15] was conducted between October 2008 and October 2009. Detailed survey methods can been found in their technical report [16]. The survey used a multi-stage, stratified, probability-proportional-to-size sample design. In brief, a three-step selection process was used to recruit participants. First, 607 representative geographical areas (mesh blocks) were defined. Then, households were randomly selected within the mesh blocks. Finally, all eligible adults (i.e. aged 15 years and older) in the household were listed and randomly selected. Māori (indigenous New Zealanders), Pacific peoples, and people aged below 19 years and over 70 years were over sampled to obtain sufficient numbers for meaningful sub-group analysis. The final sample (*n* = 4721) of people aged 15 years and over living in permanent dwellings in NZ enabled production of nationally representative estimates of dietary intake. Survey participants were visited in their home and they completed a questionnaire consisting of sociodemographic information, dietary habits and a 24-h diet recall, described in more detail below. Ethnicity data were self-reported using the standard Statistics NZ ethnicity question used in the NZ census [17]. In cases where respondents identify with more than one ethnicity, prioritised ethnicity was used as described by Statistics NZ [18]. The prioritisation is Māori, Pacific peoples, and NZ European and Other ethnicity (NZEO).

### 24-h diet recalls

Participants were visited in their homes by nutrition survey researchers who conducted detailed interviews and examined food packages and serving dishes to enhance the validity of results. All participants completed a single interviewer-administered 24-h multiple-pass diet recall [16]. In the first stage, participants were asked to list everything they consumed the previous day, from midnight to midnight, including foods, beverages and dietary supplements. Then, detailed descriptions (e.g. cooking method, brand and product name, and time consumed) for each item listed were obtained. After which, the estimates of amounts consumed were determined with the use of food photographs, food models, and common household items such as cups and spoons to assist in describing the volume of food and beverages consumed. Finally, the foods were reviewed in chronological order and participants checked the information recorded, so additions and changes could be made. At least 10% of the recalls were collected on a weekend day with the remaining 90% spread relatively evenly across the weekdays. A second 24-h diet recall was collected from 25% of the participants within a month of the first interview to measure intra-individual variability of intake [16].

**Fig. 1:**
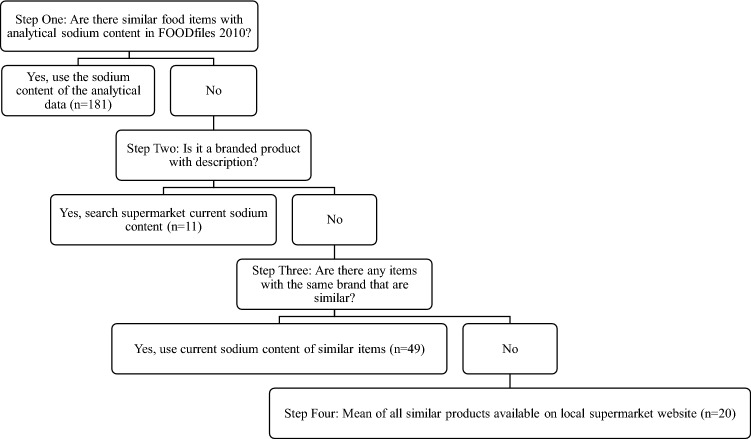
Steps to update the sodium content of 261 food items

In the 2008/09 NZANS, food items were matched to nutrient lines obtained from the NZ Food Composition Database, FOODfiles. FOODfiles is the publicly available subset of the food composition database produced by NZ Plant and Food Research, who oversee the laboratory analysis of the nutrient content of the most commonly consumed foods in NZ [19]. For foods not available within FOODfiles, a standard method for developing recipes was used to create the comprehensive 2008/09 NZANS database, where appropriate nutrient retention factors and moisture yield were applied [16]. Since dietary sodium was not considered a priority nutrient of interest when conducting the 2008/09 NZANS 24-h diet recalls [15], salt was not always added to recipes. For example, pies, crumbed chicken and battered fish. As a result, we updated the sodium content (mg/per 100 g) of 261 foods in the database using a process outlined in Fig. 1.

### Allocating foods to the HeartSAFE targets

A list of all HeartSAFE 2020 targets (Table 2) was obtained from the NZ Heart Foundation website [13]: 17 food categories and 35 sub-categories contain targets for sodium. These were mapped to foods in the NZ Food Composition Table. Where there was not an obvious match, (e.g. powdered meal bases including packet pasta and sauce dish), further information was sought from the NZ Heart Foundation. A few foods (*n* = 3) were not included in the analysis because they were not used by participants in accordance with the HeartSAFE definition of that food sub-category. For example, a soup mix was used as a seasoning but was not consumed as a soup. In total, we mapped 840 foods out of 5196 food items to the HeartSAFE sub-categories (Supplementary table 1). The updated sodium content was then merged with the 24-h diet recall data and the raw data checked for errors. Ten foods were replaced in the 24-h diet recall with the prepared version made with added liquid to meet the food sub-category definition (e.g. dried soup to soup).

### Simulation of sodium reduction

As this is presently the only sodium reduction programme available in NZ, we considered the scenario for maximum impact if all foods included in HeartSAFE 2020 met the current sodium target assigned to their respective food category. Each of the 840 foods were, therefore, assigned two sodium contents; the first was that in the 2008/09 NZANS Food Composition Table (baseline) and the second was the HeartSAFE target (simulated) [13]. 24-h diet recall data were then merged with the 2008/09 NZANS Food Composition Table to generate the baseline sodium intake and then with the HeartSAFE target to generate the simulated sodium intake; foods that were not in the HeartSAFE 2020 target categories were excluded from the analysis.

For each food sub-category, mean sodium content per 100 g at baseline was reported. To determine the most frequently consumed foods, the number of eating occasions (e.g. bread eaten at 9 am and 3 pm were considered two eating occasions) was summed for each food sub-category. The mean sodium intake per day at baseline was calculated using the amount consumed multiplied by the sodium content. Under the simulated HeartSAFE 2020 scenario, the mean simulated sodium intake per day was calculated in the same way as the baseline sodium take, except using the HeartSAFE targets. Finally, the mean reduction in sodium intake per day was estimated by subtracting the simulated sodium intake from the baseline sodium intake.

The total sodium intake from those foods that were included in the HeartSAFE 2020 food categories, for baseline and in the HeartSAFE 2020 scenario, were summed for each participant for each individual 24-h recall. The second 24-h diet recall was subsequently used to determine usual sodium intakes using the multiple source method [20]. The reduction in sodium intake per day was calculated for each participant by subtracting the usual sodium intake in HeartSAFE 2020 scenario from the baseline usual sodium intake per participant. In addition, the percentage of sodium reduction from these foods was determined by dividing the reduction in sodium intake by baseline usual sodium intake and multiplying by 100.

### Statistical analysis

All analyses were performed using Stata 13.0 [21]. For population level estimates, sampling weights were applied. They must be used when making population estimates from this complex sample, where some population groups (Māori, Pacific and some age groups) were over sampled to obtain sufficient numbers for meaningful sub-group analysis. Mean usual sodium intake was reported by sex, age group and ethnicity for baseline and HeartSAFE target scenarios. Reduction in sodium intake (mg and percent) was also calculated. Data are presented as means and 95% confidence interval.

## Results

The demographic information of the survey participants (*n* = 4721) is described in Table 1.

**Table 1:** Description of 2008/09 NZANS participants

	*n*	%
Sex		
Male	2066	43.8
Female	2655	56.2
Age group (years)		
15–18	699	14.8
19–30	718	15.2
31–50	1344	28.5
51–70	895	19.0
71 +	1065	22.6
Ethnicity		
Māori	1040	22.0
Pacific	701	14.9
NZEO	2980	63.1
Total	4721	

*NZEO* New Zealand European and Other

### Sodium reduction by food category

This analysis is restricted to food items contained in the 17 categories and 35 sub-categories targeted by HeartSAFE 2020. Baseline sodium concentrations and HeartSAFE target sodium concentrations are listed in Table 2.

**Table 2:** HeartSAFE 2020 sodium target levels and baseline mean sodium content as estimated from the 2008/09 NZANS; number of eating occasions; mean baseline sodium intake (from the 2008/09 NZANS), mean simulated sodium intake (mg/person/day), mean sodium reduction (mg/capita/day) if HeartSAFE 2020 targets met for each food sub-category^a^

HeartSAFE 2020 sodium targets categories and sub-categories		Mean (SD) mg/100 g		*n*	Mean (SD) mg/person/day		
Food category	Sub-category	Baseline sodium content^b^	HeartSAFE 2020 sodium target^c^	Eating occasions^d^	Baseline sodium intake^e^	Simulated sodium intake^f^	Simulated reduction in Na intake^g^
Bread	Leavened bread	462 (62)	380	6067	526 (389)	431 (311)	95 (101)
	Unleavened bread	351 (69)	450	143	423 (463)	407 (424)	16 (64)
Breakfast cereals	Puffed rice and corn flakes	549 (299)	500	381	177 (161)	129 (100)	47 (80)
	Oat-based muesli, porridge	111 (114)	200	309	76 (111)	62 (78)	14 (42)
	Biscuits	565 (85)	300	913	241 (142)	128 (72)	113 (73)
	Other ready-to eat cereals	421 (254)	400	545	172 (153)	130 (115)	43 (68)
Processed meats	Sausages	882 (384)	650	817	946 (931)	799 (814)	147 (321)
	Bacon	1589 (460)	1090	394	789 (650)	547 (461)	242 (290)
	Ham	1409 (109)	1090	613	910 (868)	706 (682)	204 (209)
Savoury pies	Mince/steak	485 (66)	400	226	856 (389)	703 (296)	153 (144)
	Mince and cheese/steak and cheese	441 (49)	400	222	941 (453)	848 (379)	93 (129)
Soups	All soups	330 (130)	280	263	992 (693)	782 (457)	211 (342)
Cheese	Cheddar and cheddar-style	728 (51)	710	1205	291 (284)	279 (270)	12 (20)
	Mozzarella	527 (0)	550	18	202 (185)	202 (185)	0 (0)
	Processed	1143 (201)	1270	150	371 (217)	361 (213)	10 (35)
Savoury snacks	Potato and other vegetable crisps	646 (227)	520	428	343 (343)	251 (232)	92 (144)
	Extruded/pelleted	1067 (251)	770	92	430 (466)	327 (370)	103 (108)
	Sheeted/reformed	621 (126)	520	165	356 (387)	294 (310)	62 (118)
	Popcorn	376 (231)	390	58	219 (235)	161 (170)	57 (65)
	Salt and vinegar	733 (23)	740	3	156 (108)	156 (109)	0 (1)
Gravies and sauces	Cooking sauces	447 (88)	380	572	871 (837)	703 (644)	168 (233)
	Asian sauces	4143 (2316)	680	340	687 (799)	141 (172)	546 (719)
	Gravies and finishing sauces	548 (167)	450	283	378 (312)	318 (257)	60 (103)
Powdered meal bases	Powdered meal bases	969 (0)	5000	56	3214 (1402)	3214 (1402)	0 (0)
Edible oil spreads	Margarine/oil-based spreads	433 (91)	400	4411	80 (79)	70 (64)	10 (23)
Savoury crackers	Plain	660 (155)	610	406	167 (162)	143 (136)	24 (39)
	Flavoured	757 (181)	800	251	231 (257)	211 (216)	20 (53)
	Rice and corn	499 (172)	610	195	106 (165)	99 (152)	7 (17)
Table sauce	Tomato	615 (0)	680	693	174 (182)	174 (182)	0 (0)
Canned baked beans	Canned baked beans	463 (9)	350	140	965 (619)	727 (462)	238 (159)
Canned spaghetti	Canned spaghetti	356 (23)	350	144	711 (437)	695 (425)	15 (60)
Crumbed and battered proteins	Meat and poultry	412 (220)	450	243	550 (484)	458 (384)	92 (152)
	Seafood	361 (134)	270	275	690 (489)	553 (416)	136 (189)
Ready meals	Ready meals	543 (262)	250	76	1345 (1064)	636 (398)	710 (805)
Pizzas	Pizzas	540 (100)	450	99	1177 (980)	954 (776)	222 (288)

^a^Small discrepancies may be present due to rounding

^b^Mean sodium content (mg/100 g) of each food sub-category in the food composition database

^c^HeartSAFE 2020 target published by Heart Foundation New Zealand (see Supplementary Table)

^d^Total number of times each food sub-category was consumed

^e^Mean baseline intake of sodium for each food sub-category per 24-h recall, baseline sodium content X amount consumed

^f^Mean simulated sodium intake per 24-h recall, if baseline above target, HeartSAFE 2020 target X amount consumed

^g^Mean sodium reduction (baseline sodium intake—simulated intake) for each food sub-category per 24-h recall

The most frequently consumed food sub-categories were (in decreasing order) leavened bread, margarine/oil-based spreads, cheddar and cheddar-style cheese, breakfast biscuits (e.g. “Weet-Bix”) and sausages.

The top five food sub-categories that had the highest reduction in sodium were ready-meals (710 mg/person/day), Asian sauces (546 mg/person/day), bacon (242 mg/person/day), canned baked beans (238 mg/person/day), and pizzas (222 mg/person/day). Out of the 35 food sub-categories, these high sodium reduction sub-categories ranked 31st, 14th, 12th, 28th, and 29th most frequently consumed items.

Conversely, there was little or no reduction in the sodium content of mozzarella cheese, processed cheese, powdered meal bases, and tomato sauce; most of these foods already met the target.

### Population level sodium consumption

Table 2 shows the estimated population usual sodium intake from the food categories included in HeartSAFE 2020 before and after applying the targets. Mean sodium intake from these foods was 1307 mg/day at baseline and 1048 mg/day for the simulated HeartSAFE 2020 scenario. This represents a 260 mg/day or 20% reduction in sodium intake from these foods.

The mean baseline intake of sodium from foods included in this analysis for men was 1464 mg/day and 1162 mg/day for women. The mean simulated sodium reduction was 288 mg/day for men and 233 mg/day for women. The estimated sodium reduction ranged from 281 mg/day in the 19–30 years old group to 233 mg/day in those 71 + years old. Sodium intake from included foods in Māori, Pacific and NZ European and Other (NZEO) ethnicities was 1399 mg/day, 1292 mg/day and 1296 mg/day, respectively, with a reduction in sodium intakes ranging from 258 to 270 mg/day (Table 3). Sodium reduction from included foods was between 19 and 21% across all population groups.

**Table 3:** Population estimates for sodium intake at baseline and simulated reduction assuming HeartSAFE 2020 targets met in all foods category included, mean absolute reduction (mg/day) in total and by sex, age group and ethnicity^a,b^

	*n*	Mean (95% CI) baseline sodium intake, mg/day	Mean (95% CI) simulated sodium intake based on HeartSAFE 2020 targets, mg/day	Mean (95% CI) absolute sodium reduction, mg/day	Mean % sodium reduction (95% CI)
Total	4721	1307 (1279, 1336)	1048 (1024, 1072)	260 (252, 268)	20 (19, 20)
Sex					
Male	2066	1464 (1412, 1516)	1176 (1133, 1219)	288 (274, 303)	20 (19, 20)
Female	2505	1162 (1131, 1193)	929 (903, 954)	233 (224, 243)	20 (19, 20)
Age group (years)					
15–18	699	1365 (1295, 1434)	1100 (1043, 1157)	265 (248, 282)	19 (19, 20)
19–30	718	1360 (1282, 1438)	1079 (1014, 1145)	281 (254, 307)	21 (19, 22)
31–50	1344	1362 (1310, 1413)	1090 (1049, 1130)	272 (257, 287)	20 (19, 20)
51–70	895	1220 (1166, 1274)	985 (940, 1029)	236 (223, 249)	19 (19, 20)
71 +	1065	1191 (1154, 1227)	958 (931, 986)	233 (220, 245)	19 (19, 20)
Ethnicity					
Māori	1040	1399 (1326, 1472)	1129 (1067, 1192)	270 (254, 285)	20 (19, 20)
Pacific	701	1292 (1223, 1362)	1026 (971, 1081)	266 (245, 288)	20 (19, 20)
NZEO	2980	1296 (1263, 1329)	1038 (1011, 1065)	258 (248, 268)	20 (19, 20)

*CI* confidence interval; *NZEO* New Zealand European and Other

^a^Small discrepancies may be present due to rounding

^b^Only applies to sodium intake from foods included in the HeartSAFE 2020 targeted food categories and does not represent all sodium intake

## Discussion

Using the 2008/09 NZANS, we simulated the potential reduction in sodium intake in adults if the sodium content of all 840 foods in 17 food categories and 35 sub-categories met the HeartSAFE 2020 voluntary targets. These data were collected prior to the implementation of the HeartSAFE sodium reduction programme [22], which allowed our study to estimate the maximum sodium reduction from the time of implementation until the present. The foods targeted by the HeartSAFE programme contributed substantially to sodium intake. Our estimates show that these foods contributed to around 1300 mg/day of sodium intake at baseline, roughly 40% of total intake based on recent studies [5–7]. However, our results demonstrated that when the HeartSAFE 2020 targets were applied, this reduced mean adult sodium intake for these foods by 260 mg/day. This was equivalent to a 20% reduction in sodium intake from the targeted foods, but was a small proportion of total sodium intake, estimated to be 3100–3500 mg/day [5–7]. Based on previous studies [5–7], we estimate that a reduction of 260 mg/day via the HeartSAFE 2020 programme would reduce total sodium intake by less than 8%. Similar results are obtained if total sodium intake is estimated from spot urine (i.e. 8.5%) or 24-h diet recall (i.e., 10%) data from the 2008/09 NZANS [23]. This falls well short of the 30% sodium reduction target set by WHO [1]. Furthermore, our estimate assumed that all HeartSAFE 2020 targets were met, but these targets are voluntary, thus the overall impact of the programme on total sodium intake is likely to be even lower.

Our study found item such as cheese, amongst food categories with the least sodium reductions. Previous research has reported reduction in sodium content of cheese being poorly received by consumers [24, 25]. Furthermore, there are technological difficulties in reducing sodium in cheese production. Salt is required for maintaining safety and quality such as inhibiting microbial growth, regulating water and enzyme activity and influencing flavour and aroma [26]. On the other hand, meat-based items are granted a wider margin for sodium reduction before consumer acceptability is affected [24, 25]. Technologically, reduced-sodium meat products are also more feasible owing to the use of spices and phosphates [26]. In soups, where salt is added mainly for flavour [27], salt can be reduced up to 48% while retaining purchase intent and consumer acceptability [28]. Incidentally, these are also items that had sodium reduced to a larger extent in our study.

Our findings show that food reformulation of a limited range of foods, alone, is insufficient to achieve meaningful sodium reduction, a finding that aligns with other studies undertaken in different countries. Dunford and Poti simulated the reduction in sodium intake in pre-packaged foods using the 2010/2011 US National Health and Nutrition Examination Survey (NHANES) [29]. In their analysis, if the sodium content of all packaged foods were reduced from the 50th to the 25th percentile, sodium intake in adults from packaged foods would decrease by 13.3% or 167 mg/day. They also found sodium intake reduction was different between ethnic groups in the USA. Hispanic adults reduced sodium intake by 12.9% while Non-Hispanic White and Non-Hispanic Black by 13.3% and 13.7%, respectively. In contrast, we found that the reduction in sodium intake, if all foods included in HeartSAFE 2020 met the target, would be similar across ethnic groups in NZ. This indicates that the HeartSAFE 2020 targets could improve sodium intake in all groups across the population in NZ. Using the national French dietary survey (INCA2) conducted in 2006/07, dietary modelling was undertaken by applying sodium targets for 21 food groups in the International Choices Programme [30]. The study reported a 12.7% reduction in sodium intake from these foods. In the USA, a longitudinal study from 2000 to 2014 examined the change in sodium intake from packaged foods using food purchase data (Nielsen Homescan Consume Panel), reporting a reduction of 260 mg/day [31]. This study has a number of parallels to our findings, including the timeframe of the study (i.e. > 10 years) and the amount of sodium reduced (i.e. both 260 mg/day). In the USA, the National Salt Reduction Initiative (NSRI) develops targets to guide the voluntary reformulation programme, and although the USA targets for sodium content are slightly higher than that of HeartSAFE 2020, the number of products included (62 categories) is more extensive than the NZ HeartSAFE programme [32]. Another recent Australian study, collected food purchased data (Nielsen Homescan Consume Panel) in 2018 [33]. Similar to our study, they simulated a mandatory scenario for sodium reduction in 27 food categories included in their Healthy Food Partnership programme. Their findings were more disheartening as they suggested that sodium intake would only reduce by 50 mg/day if all foods met the target in the programme.

Federici and colleagues conducted a systematic review to examine the effects of food reformulation on reducing the intakes of saturated fat, added sugar, and sodium [34]. The study included peer-reviewed articles published between January 2000 and December 2017 of modelling studies of foods commonly sold in retail stores. Of the 33 studies included, sodium was the most commonly targeted nutrient for food reformulation modelling (*n* = 25), primarily in bread, sauces and processed meats. The reduction in sodium content in these foods ranged from 11 to 63%. There was a wide range of sodium targets in included studies, and the degree of sodium reduction was proportional to the percentage of sodium reformulated. Of the studies where absolute reduction in sodium intake was reported (*n* = 24), this ranged from 9 to 1820 mg/day/person [34].

The WHO *SHAKE Technical Package for Salt Reduction* recommends that countries implement a range of measures to achieve meaningful salt reduction. These include reformulation of processed foods and prepared meals, improvements in labelling and marketing of foods, educating consumers, and environmental measures to support healthy eating [35]. A previous NZ study has estimated that a 30% reduction in sodium intake would require a 36% reduction in sodium across a wide range of packaged foods, a 40% reduction in takeaway and restaurant meals, as well as a 40% reduction in discretionary salt use (salt added in the home in cooking and at the table) [36]. This is consistent with the recommendations in the SHAKE Technical Package [4].

Our study adds to the growing body of evidence demonstrating that isolated voluntary strategies are unlikely to make a meaningful impact on total sodium intakes. Currently in NZ, sodium reduction largely depends on the HeartSAFE programme and the Health Star Rating system, a nutrient-based signpost front-of-pack labelling system. The Health Star Rating System provides an overall rating (in the form of number of stars out of 5) of how healthy a food is rated based on a nutrient profiling system [37]. While it includes sodium in the algorithm, it does not enable consumers to identify low-sodium foods specifically, unlike the Traffic light system adopted by other countries. Previous research has indicated that a traffic light system that includes a sodium-specific traffic light would help New Zealand consumers to limit sodium intake [38]. Further, both the HeartSAFE and Health Star Rating System are voluntary in nature and endorsed by the food industry [39]. In 2003/04, the United Kingdom (UK) initiated one of the most successful sodium reduction campaigns in the world, to date, achieving a 15% reduction in 24-h urinary sodium by 2011 [40]. As part of a comprehensive strategy that included consumer education and improvements in food labelling, the UK Food Standards Agency engaged with the food industry to develop new food reformulation targets. The UK targets included a much wider range of foods and with lower sodium contents than the NZ HeartSAFE programme. A NZ simulation study used the UK targets with supermarket sales data found that reformulation in packaged foods resulted in a sodium reduction of 628 mg/day, double our estimated reduction [36].

Recently, experts have called for a re-think of the nutrient-to-limit model of food reformulation which has a narrow focus on one nutrient rather than across the whole diet [41, 42]. Reformulation reduces the negative impact of mainly ultra-processed foods, but does not promote the shift to nutritionally superior, minimally processed foods, such as fruits, vegetables, seeds, and nuts [43]. In addition to food reformulation, there are other policies and interventions that have been implemented elsewhere and could be employed in NZ. Examples include imposing tax on products with high sodium contents (Portugal), regulating nutrition health claims (European Union), front-of-pack labelling (warning labels on high sodium products, Chile) and interventions in public institutions (schools, universities, hospitals, and workplaces) involving nutrition education and sodium standards in foods sold or catered in these institutes [25, 43–45]. Academic experts in NZ have urged the government to take the lead and implement such policies, including mandatory food reformulation, food marketing, food labelling, healthier retail environments, monitoring and evaluation, and health-protecting taxation [46, 47]. These recommendations are in line with the international shift towards a more holistic approach to improving food environments which is also signalled in the WHO SHAKE technical package.

A major strength of this study is the nationally representative nature of our dataset which enables us to evaluate the potential reduction of sodium intake at a population level. Another strength is that the data were collected at the start of the HeartSAFE programme, which allows us to estimate the impact of the programme. It is possible that food habits have changed over the last 13 years; indeed, there is evidence to suggest that bread consumption has fallen [48] and intakes of fast food have increased [49]. However, HeartSAFE has not set sodium targets for fast foods, so this would not have altered our findings. Since sodium was not a nutrient of interest in the 2008/09 NZANS, we had to update the sodium content of some recipes. This was done carefully in a systematic manner. Under-reporting can occur with 24-h diet recalls, especially of foods that are considered unhealthy, such as snacks, that might also be high in sodium [50]. Although dietary assessment may have measurement errors, it is not without its merits [51], particularly for this study, as we were able to identify individual foods and to alter the sodium content corresponding to the HeartSAFE 2020 target. Indeed, WHO recommends dietary assessment data to be used as the first source of data input in sodium simulation studies such as ours [52]. In our estimation of the overall impact of the HeartSAFE programme using total sodium intake estimated from 24-h urine in previous studies or 24-h diet recall and spot urine from 2008/09 NZANS data, the results were similar.

## Conclusion

Excess sodium intake is detrimental to health. Food reformulation programmes are currently the main driver of sodium reduction initiatives in NZ, however, they are limited to a small number of foods, and are voluntary. This study shows that the current sodium targets featured in the NZ HeartSAFE programme will not meet the 30% sodium intake reduction set out by the WHO Global Action Plan. A more comprehensive strategy consistent with the WHO SHAKE technical package is needed to advance the goal of sodium intake reduction.

## Supplementary Information

Below is the link to the electronic supplementary material.

Supplementary file1 Supplementary 1 Mapping of 840 foods included to the 35 sub-food categories in HeartSAFE 2020. (XLSX 189 KB)

## Data Availability

The authors received approval from the Ministry of Health New Zealand to use the 2008/09 NZANS data set (CURF2020-09).
